# Sialic Acid Receptor Specificity in Mammary Gland of Dairy Cattle Infected with Highly Pathogenic Avian Influenza A(H5N1) Virus

**DOI:** 10.3201/eid3007.240689

**Published:** 2024-07

**Authors:** Rahul K. Nelli, Tyler A. Harm, Chris Siepker, Jennifer M. Groeltz-Thrush, Brianna Jones, Ning-Chieh Twu, Ariel S. Nenninger, Drew R. Magstadt, Eric R. Burrough, Pablo E. Piñeyro, Marta Mainenti, Silvia Carnaccini, Paul J. Plummer, Todd M. Bell

**Affiliations:** Iowa State University College of Veterinary Medicine, Ames, Iowa, USA (R.K. Nelli, T.A. Harm, C. Siepker, J.M. Groeltz-Thrush, B. Jones, N.-C. Twu, A.S. Nenninger, D.R. Magstadt, E.R. Burrough, P.E. Piñeyro, M. Mainenti, P.J. Plummer, T.M. Bell);; University of Georgia, Athens, Georgia, USA (S. Carnaccini)

**Keywords:** sialic acid, influenza, viruses, Neu5Ac, α2,3-gal, α2,6-gal, H5N1, highly pathogenic avian influenza, dairy cattle, mammary gland, epithelial cell, macrophage, respiratory tract, zoonoses, United States

## Abstract

In March 2024, the US Department of Agriculture’s Animal and Plant Health Inspection Service reported detection of highly pathogenic avian influenza (HPAI) A(H5N1) virus in dairy cattle in the United States for the first time. One factor that determines susceptibility to HPAI H5N1 infection is the presence of specific virus receptors on host cells; however, little is known about the distribution of the sialic acid (SA) receptors in dairy cattle, particularly in mammary glands. We compared the distribution of SA receptors in the respiratory tract and mammary gland of dairy cattle naturally infected with HPAI H5N1. The respiratory and mammary glands of HPAI H5N1–infected dairy cattle are rich in SA, particularly avian influenza virus–specific SA α2,3-gal. Mammary gland tissues co-stained with sialic acids and influenza A virus nucleoprotein showed predominant co-localization with the virus and SA α2,3-gal. HPAI H5N1 exhibited epitheliotropism within the mammary gland, and we observed rare immunolabeling within macrophages.

The recent discovery that dairy cattle can be infected by highly pathogenic avian influenza (HPAI) virus of the H5N1 subtype (*1*,*2*), in combination with the virus’s propensity to replicate in the mammary gland, has raised many questions and is causing fresh concerns about HPAI H5N1 spread (*3*). Detection of high levels of viral RNA in the milk during the acute stage of infection (*1*) has triggered public health alerts and premovement testing requirements (*4*), and has suggested the potential for the presence of the virus in unpasteurized milk.

HPAI H5N1 clade 2.3.4.4b virus was first detected in wild birds in the United States in late 2021 (5–7) and spread widely across North America (*7*,*8*). In early 2022, HPAI H5N1 virus began causing outbreaks in commercial and backyard poultry flocks. The US Department of Agriculture’s Animal and Plant Health Inspection Service reported >6,400 cases in wild birds in 49 states and >790 backyard flocks in 47 states during January 2022–March 2023 (*7*). The widespread distribution of HPAI H5N1 virus eventually spilled into multiple mammal species within those areas, often presumably because of the consumption of infected wild birds (*8*). By the end of 2023, HPAI H5N1 had been reported in >20 different mammal species in the United States (*9*). Dairy cattle were affected in the spring of 2024, when multiple dairy herds were determined to be positive for HPAI H5N1 clade 2.3.4.4b virus (*1*,*2*). On the basis of genetic sequencing, this introduction into dairy cattle is thought to be from a wild bird source (*1*,*2*).

A major determinant of a virus-host range is receptor availability (*9*,*10*). Influenza A viruses (IAVs) use host sialic acids as their receptors for initial attachment and entry into the cells. Sialic acids (SAs) are a diverse group of 9-carbon carboxylated monosaccharides synthesized in animal species. IAVs from avian species have been shown to preferentially bind to SA receptors linked to galactose by an α2,3-galactose linkage (SA α2,3-gal). More specifically, IAVs from chickens preferentially bound to SA α2,3-gal-β (1–4) N-acetylglucosamine (GlcNAc), whereas IAVs from ducks displayed a higher affinity for SA α2,3-gal-β (1–3) N-acetylgalactosamine (GalNAc) (*10*). IAVs originating from human and classical swine viruses prefer SA receptors with an α2,6-galactose linkage (SA α2,6-gal) (*10*–*14*). Various methods have been used to characterize the SA receptor distribution profiles among multiple host species. Plant lectin binding affinity toward SA is used to study SA distribution in animal tissues (*15*). Plant lectins from the species *Sambucus nigra* (SNA) are routinely used to detect α2,6-linked SA; *Maackia amurensis* lectin–I (MAL-I) is used to detect SA α2,3-gal-β (1–4) GlcNAc and *M. amurensis* lectin–II (MAL-II) to detect SA α2,3-gal-β (1–3) GalNAc.

We explored the presence and distribution of cellular and receptor factors that enable HPAI H5N1 virus infection in Holstein dairy cattle. The previously reported index cases highlighted a multifocal or segmental distribution of IAV within provided mammary gland samples (*1*). This type of distribution was of particular interest regarding potential binding sites within the lactiferous tree. Specifically, our study reports the expression and distribution of SA receptors using lectin histochemistry and viral binding assay in the bovine respiratory tract and mammary glands of dairy cattle that were affected with HPAI H5N1 clade 2.3.4.4b virus (A/dairy_cattle/Texas/24_009110). Our study seeks to uncover the underlying reasons behind the uncommon mammary gland infection by HPAI H5N1 virus.

## Materials and Methods

### Sample Collection

We used formalin-fixed and paraffin-embedded sections of trachea, lung, and mammary gland tissues, as well as milk in EDTA tubes, from 2 adult Holstein dairy cows in Texas, USA, diagnosed with HPAI H5N1 virus infection at the Iowa State University Veterinary Diagnostic Laboratory (Ames, IA, USA) on March 2024 (*1*). The cows reportedly exhibited a nonspecific illness, including reduced lactation and thickened, yellow milk with a similar appearance to colostrum. The diagnosis was based on detection of HPAI H5N1 IAV by real-time reverse transcription PCR (rRT-PCR) in the mammary gland and lung, as well as by detecting IAV nucleoprotein by immunohistochemistry in the mammary glands (*1*). In addition, we confirmed affected tissue sections to be positive for IAV matrix gene nucleic acid by RNAscope in situ hybridization assay (Appendix Figure 1). Testing of macroscopic and microscopic lesions, including IAV chromogenic immunohistochemistry and rRT-PCR, was previously described (*1*). The mammary glands from the cows had a multifocal lesion pattern, which enabled the dissection of affected and unaffected regions of the mammary gland. We used unaffected regions as control tissue. To be classified as unaffected, the sections could not have any inflammation or epithelial changes. In addition, unaffected sections were required to have a negative IAV immunohistochemistry result.

### Cytologic Evaluation of Milk Sample

We used milk from EDTA tubes to make direct smears onto cytocentrifuged slides. We performed cytocentrifugation of milk by using a Shandon Cytospin3 (ThermoFisher Scientific, https://www.thermofisher.com) at 72 g for 10 minutes (low acceleration). We prepared air-dried slides and stained them with modified Wright stain on an automated stainer (Siemens Healthineers, https://www.siemens-healthineers.com). We determined differential cell counts under ×1,000 original magnification by counting 100 nucleated cells.

### Fluorescently Labeled Dual Lectin and Immunochemistry Staining

We characterized mammary tissues for sialic acids by using an automated adaptation of a lectin histochemistry assay previously described (*16*) combined with influenza A nucleoprotein (IAV-Np) staining. We sectioned formalin-fixed, paraffin-embedded tissue sections of the bovine mammary gland at 4 μm with placement on Superfrost Plus slides (VWR International, https://www.vwr.com). We dried slides at 60°C for 20 minutes before deparaffinization and staining on the Ventana Discovery Ultra research platform (Roche Diagnostics, https://diagnostics.roche.com). We accomplished heat retrieval by using cell conditioning solution at 100°C for 24 minutes (Roche Diagnostics). We blocked slides with 1X Carbo-Free blocking solution (Vector Laboratories, https://vectorlabs.com) for 32 minutes, then used a Streptavidin/Biotin Blocking Kit (Vector Laboratories) with a separate application each of 12 minutes. We incubated the sections for 4 hours at room temperature with 1 of the 3 lectins (SNA, MAL-I, MAL-II) from Vector Laboratories at the specified concentrations (Appendix Table) (*17*), where SNA is specific for α2,6-gal/GalNAc, MAL-I is specific for N-linked or O-linked glycans with SAα2,3-gal-β (1–4) GlcNAc, and MAL-II is specific for O-linked glycans with SA α2,3-gal-β (1–3) GalNAc. After lectin incubation, we applied streptavidin conjugated with Alexa Fluor 647 (ThermoFisher Scientific) and incubated the sections for 2 hours at room temperature. We performed immunostaining with IAV-Np by incubating sections with rabbit recombinant monoclonal anti-nucleoprotein for IAV labeled with DyLight 594 (Novus Biologicals, https://www.novusbio.com) for 4 hours at room temperature. We manually performed counterstaining and mounting with Prolong Gold antifade mountant with DAPI (ThermoFisher Scientific). Negative assay controls consisted of primary lectin or antibody omission. We evaluated nonspecific lectin labeling by using sialidase-A treated sections (Appendix). We performed positive lectin assay controls on porcine tissues because lectin labeling has been previously established (*17*). We also performed positive IAV-Np controls on porcine lung tissue with known IAV infection status (data not shown). We performed multicolor fluorescent staining by using lectin and IAV-Np assay to aid co-labeling.

We conducted multicolor immunofluorescent staining by using mouse monoclonal anti-human cytokeratin, Clone AE1/AE3 (Agilent Technologies, https://www.agilent.com), and rabbit monoclonal anti-ionized calcium binding adaptor molecule 1 (Iba1) (Abcam, https://www.abcam.com), along with the primary IAV-Np antibody previously described (Appendix Table). Modifications to this lectin-antibody procedure include heat retrieval with cell conditioning solution for 48 minutes at 100°C, replacing the lectin with an antibody incubation at 37°C (Iba1 at 60 minutes and cytokeratin at 32 minutes), as well as incubation of a Biotinylated Link from a Universal LSAB2 Kit (Agilent Technologies) for 1 hour before application of the streptavidin conjugate. We then examined slides and imaged them by using a BX-53 Olympus trinocular microscope equipped with an Olympus DP23 camera, Excelitas X-Cite mini+ compact illumination system, and CellSyns Dimension software.

## Results

We selected retrospectively collected respiratory tract and mammary gland samples from 2 naturally infected lactating Holstein dairy cows with HPAI H5N1 virus infections for evaluation. The clinicopathologic manifestations, detection methods, and sequencing data pertaining to the dairy cows in this study have been previously reported (*1*,*2*).

**Figure 1:**
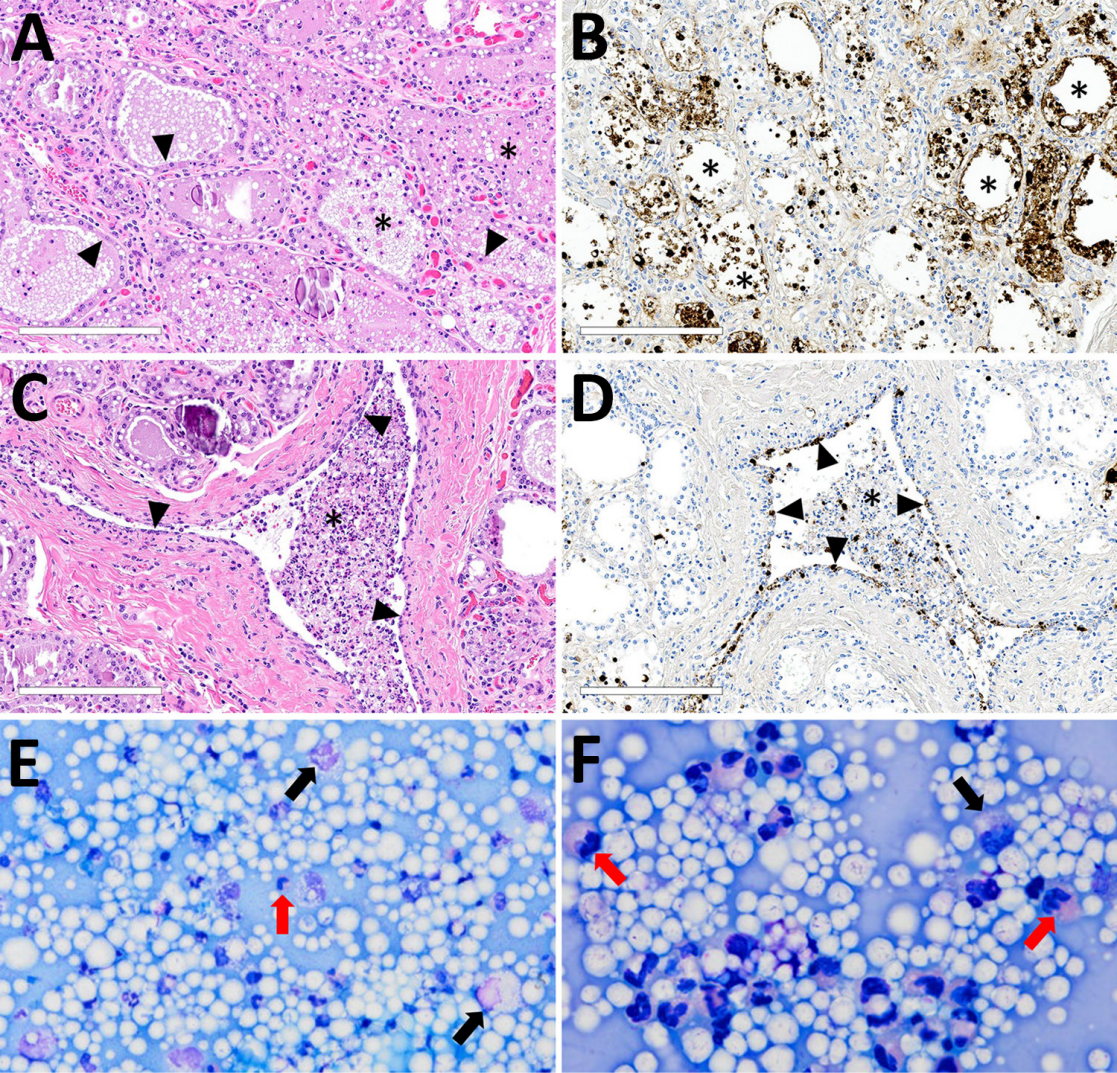
Microscopic mammary lesions of an index case in US dairy cattle naturally infected with highly pathogenic avian influenza A(H5N1) virus clade 2.3.4.4b. A) Mammary gland alveoli show epithelial attenuation and vacuolation (arrows), leading to degeneration with intraluminal sloughing and neutrophilic intraluminal inflammation (asterisk). Hematoxylin and eosin stain. B) Mammary glad alveoli show degenerative epithelial cells (asterisks) and strong intracytoplasmic and nuclear immunoreactivity to influenza A virus nucleoprotein. C) Cuboidal epithelium lining of the interlobular duct was markedly attenuated (arrowheads) with abundant intraluminal sloughing and neutrophilic inflammation (asterisk). Hematoxylin and eosin stain. D) Interlobular duct shows of attenuating and degenerative epithelium by intranuclear and cytoplasmic immunoreactivity (brown labeling) with immunopositive intraluminal debris and inflammation (asterisk). Scale bars indicate 200 μm. E, F) Modified Wright's stained representative cytology images of milk from a dairy cow with highly pathogenic avian influenza A(H5N1) virus infection, demonstrating moderate to marked neutrophilic inflammation (red arrows) with low numbers of vacuolated macrophages (black arrows) among large numbers of lipid vacuoles. A 100-cell count was performed, and nucleated cells were found to consist of 83% neutrophils, 12% macrophages, and 5% lymphocytes. Original magnifications ×500 for panel E and ×1,000 for panel F.

In brief, the HPAI H5N1 virus–infected mammary gland had acute multifocal moderate mastitis with epithelial attenuation lining the secretory alveoli and interlobular ducts, as well as intraluminal neutrophilic inflammation (Figure 1, panel A, C). Immunohistochemistry for IAV-Np on the affected mammary gland showed intranuclear and intracytoplasmic immunoreactivity in alveolar and interlobular ductal epithelial cells (Figure 1, panel B, D). We examined the milk from the affected gland cytologically, which had not previously been reported. Routine cytologic assessment identified moderate to marked neutrophilic and mild macrophagic inflammation consistent with mastitis (Figure 1, panel E, F).

We considered the evaluation of bovine tissue distribution for SA α2–3 and SA α2–6 receptors to be warranted, given the detection of HPAI H5N1 virus and lesion development in the dairy cows. We evaluated the distribution of SA α2–3 and SA α2–6 receptors in the respiratory and mammary tissues from the cows through fluorescent and chromogenic-based lectin histochemistry.

**Figure 2:**
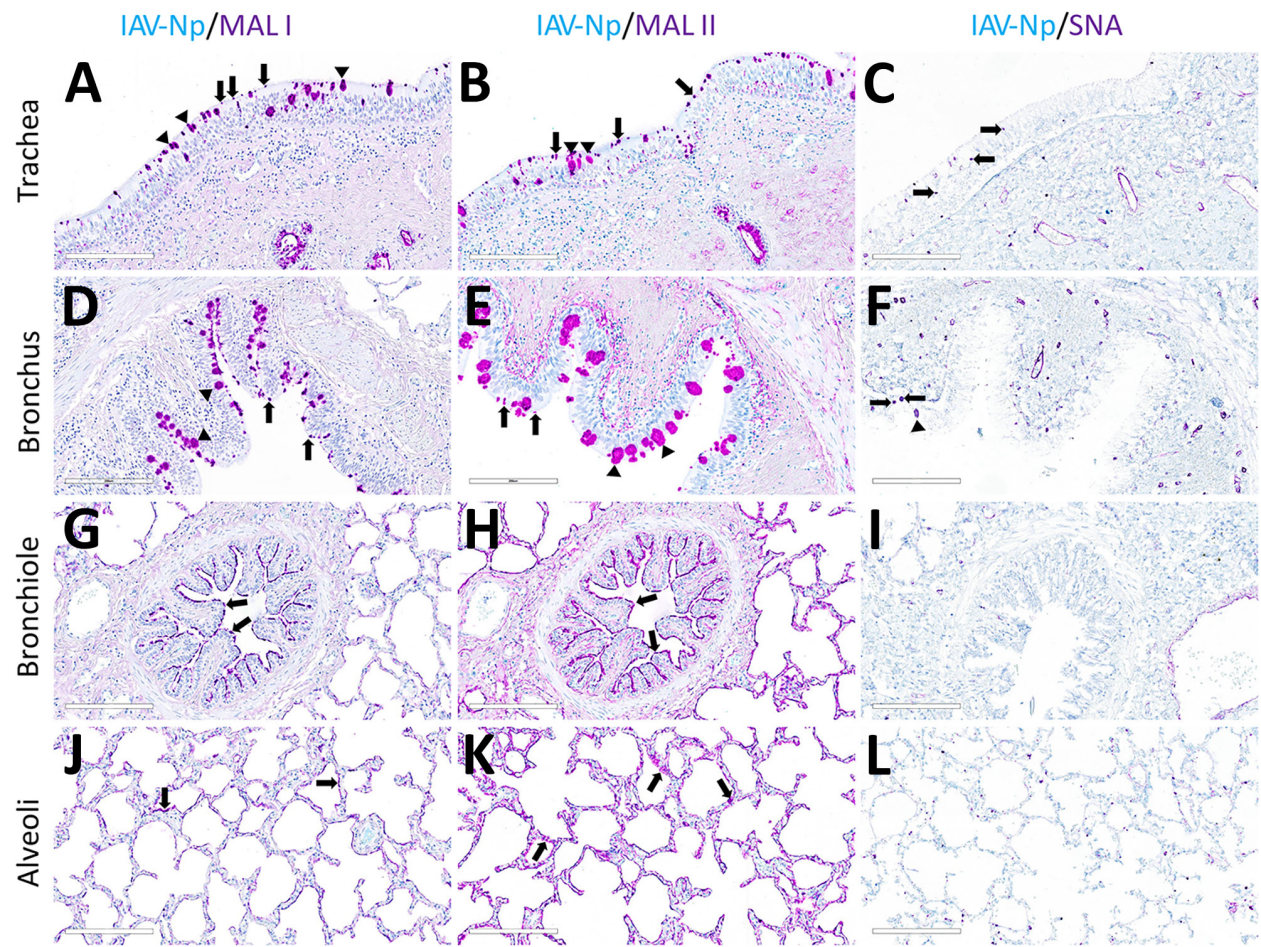
Respiratory tract tissues from a US dairy cow infected with highly pathogenic avian influenza A(H5N1) virus, showing IAV-Np (teal chromogen), individually duplexed with MAL-I (magenta chromogen), MAL-II (magenta chromogen), and SNA (magenta chromogen) using chromogenic staining. Representative images are shown for IAV-Np/MAL-I (A, D, G, J), IAV-Np/MAL-II (B, E, H, K), and IAV-Np/SNA (C, F, I, L) are shown. No IAV-Np was observed in the unaffected respiratory tissue sections. Intense granular to punctate labeling for MAL-I (A) and MAL-II (B) were observed within goblet cells (arrowheads), along the apical ciliated margin (arrows), and glands of the trachea. SNA labeling (C) was confined to intraepithelial round cells (arrows), endothelium, and glands of the trachea. Similar labeling for MAL-I (D) and MALII (E) was observed within the bronchial lumen within goblet cells (arrowheads) and along the apical cell margin (arrows). SNA (F) labeling was only observed in rare goblet cells (arrowheads) and lamina proprial round cells (arrows) in the bronchus. Bronchioles had diffuse, fine, fibrillary to apical membranous labeling (arrows) MAL-I (G) and MALII (H). No substantial labeling was detected within the mucosal epithelial cells of bronchioles with SNA (I). Diffuse, fine, apical membranous labeling of pneumocytes lining alveoli was observed with MAL-I (J) and MAL-II (K) (arrows). SNA (L) labeling within the alveolar portions of the lung was confined to endothelium and interstitial round cells. Scale bars indicate 200 μm. IAV-Np, influenza A virus nucleoprotein; MAL, *Maackia amurensis* lectin; SNA, *Sambucus nigra* lectin.

**Figure 3:**
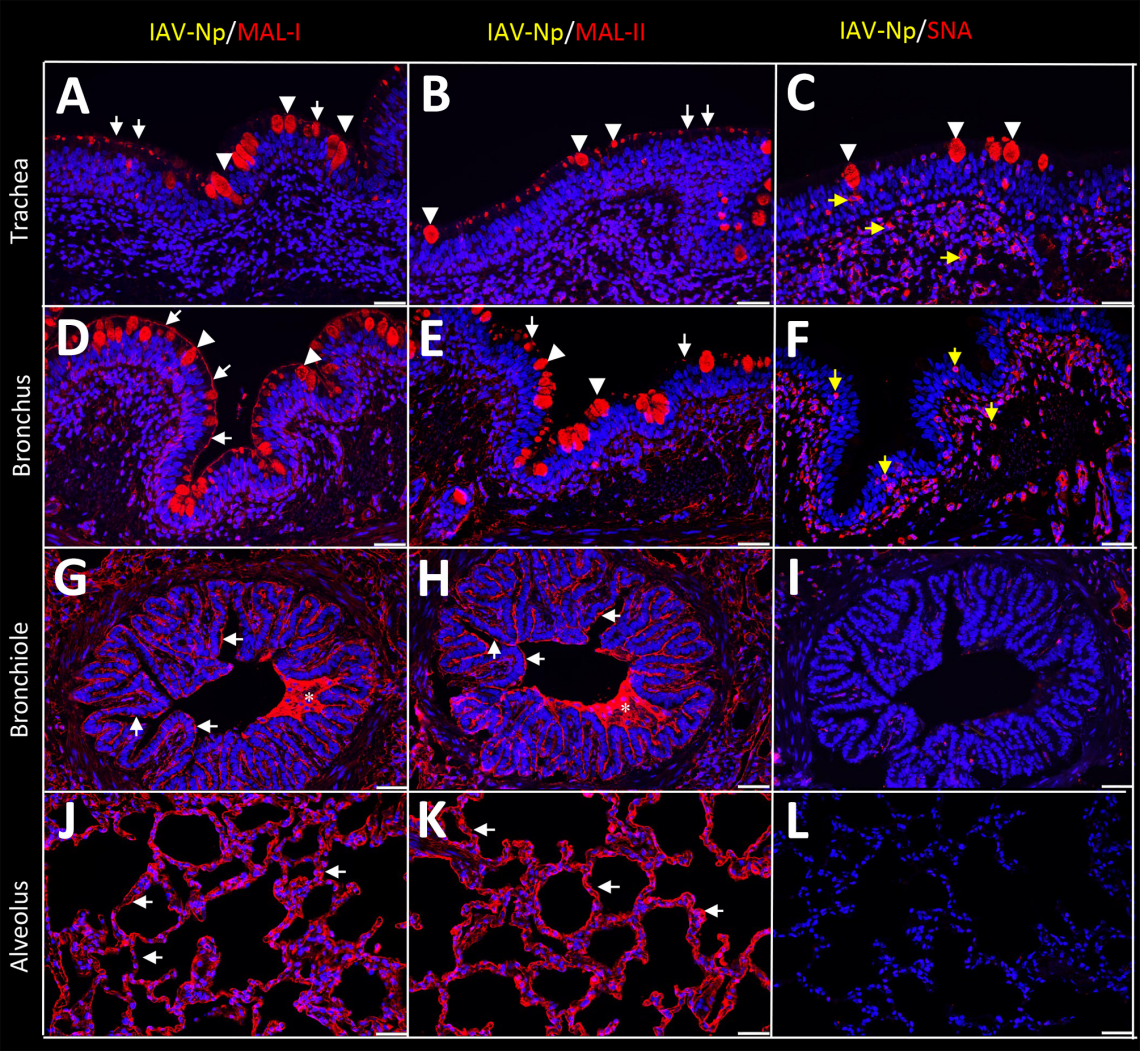
Respiratory tract tissues from a US dairy cow infected with highly pathogenic avian influenza A(H5N1) virus, labeled with IAV-Np (yellow pseudocolor, DyLight 594), individually duplexed with MAL-I (red pseudocolor, Alexa Fluor 647), MAL-II (red pseudocolor, Alexa Fluor 647), and SNA (red pseudocolor, Alexa Fluor 647) using fluorescent staining. Representative merged images are shown for IAV-Np and MAL-I (A, D, G, J), IAV-Np and MAL-II (B, E, H, K), and IAV-Np and SNA (C, F, I, L). IAV-Np labeling was not detected within the respiratory tissue sections. Intense, granular to punctate, cytoplasmic MAL-I, MAL-II, and SNA labeling was observed in goblet cells (arrowheads) and glands of the trachea (A–C). Similar goblet cell labeling (arrowheads) for MAL-I (D) and MAL-II (E) was observed in the bronchi with weak SNA labeling (F). Multifocal, moderate, fibrillar, apical, membranous MAL-I (A) and MAL-II (B) labeling (white arrows) was observed on the tracheal epithelium. The respiratory epithelium of the bronchi, bronchioles, and alveoli had diffuse, moderate to intense, apical, fibrillar MAL-I labeling (white arrows) (D, G, J). The respiratory epithelium of the bronchi had multifocal, moderate, fibrillar, apical MAL-II labeling (white arrows) (E). The respiratory epithelium of the bronchioles and alveoli had diffuse MAL-II labeling (white arrows) (H, K). Intraluminal secretory material (asterisks) in the bronchi and bronchioles were intensely labeled with MAL-I and MAL-II (G, H). Membranous, granular SNA labeling (yellow arrows) was observed in intraepithelial and lamina proprial round cells in the trachea and bronchi (C, F). Scale bars indicate 50 μm. IAV-Np, influenza A virus nucleoprotein; MAL, *Maackia amurensis* lectin; SNA, *Sambucus nigra* lectin.

Both fluorescent and chromogenic lectin histochemistry methodologies concur with the distribution of lectins (Table; Figures 2, 3). We only observed SNA labeling in goblet cells, submucosal glands, and intraepithelial and lamina proprial immune cells (suspected to be lymphocytes) of the trachea and bronchi (Figure 2; Figure 3, panels C, F). MAL-I and MAL-II labeling was multifocal, weak to moderate, apical, and membranous in the respiratory epithelium of the trachea (Figure 2; Figure 3, panels A, B). MAL-I and MAL-II labeling in the tracheal and bronchial goblet cells and submucosal glandular epithelial cells was intense, granular, and cytoplasmic (Figure 2; Figure 3, panels A, B, D, E). However, MAL-I and MAL-II labeling differed in the bronchial respiratory epithelium, where MAL-I labeling (Figure 2; Figure 3, panel D) was more diffuse, whereas MAL-II labeling (Figure 2; Figure 3, panel E) was multifocal. MAL-I (Figure 2; Figure 3, panels G, J) and MAL-II (Figure 2; Figure 3, panels H, K) labeling of the respiratory epithelium was similar in the bronchioles and alveoli, where labeling was diffuse.

**Table:** Distribution of α2,3 and α2,6 receptors with influenza A virus nucleoprotein in the respiratory tract and mammary gland of US dairy cattle naturally infected with highly pathogenic avian influenza A(H5N1) virus*

Site	MAL-I	MAL-II	SNA	IAV-Np
Respiratory tract				
Tracheal epithelium	+ (mem, goblet)	+ (mem, goblet)	+/− (goblet)	–
Bronchial epithelium	+ (mem, goblet)	+ (mem, goblet)	–	–
Bronchiolar epithelium	+ (mem)	+ (mem)	–	–
Pneumocytes, alveolus	+ (mem)	+ (mem)	–	–
Mammary gland				
Glandular epithelium	–	+ (mem)	+ (mem)	+ (IN, IC)
Interlobular ductal epithelium	–	–	+ (mem)	+ (IN, IC)

*Goblet, goblet cells; IC, intracytoplasmic; IN, intranuclear; IAV-Np, influenza A virus nucleoprotein; MAL, *Maackia amurensis* lectin; mem, membranous; SNA, *Sambucus nigra* lectin; –, no labeling observed; +, positive labeling. †α2,3-gal-β (1–4) N-acetylglucosamine. ‡α2,3-gal-β (1–3) N-acetylgalactosamine. §α2,6-gal.

**Figure 4:**
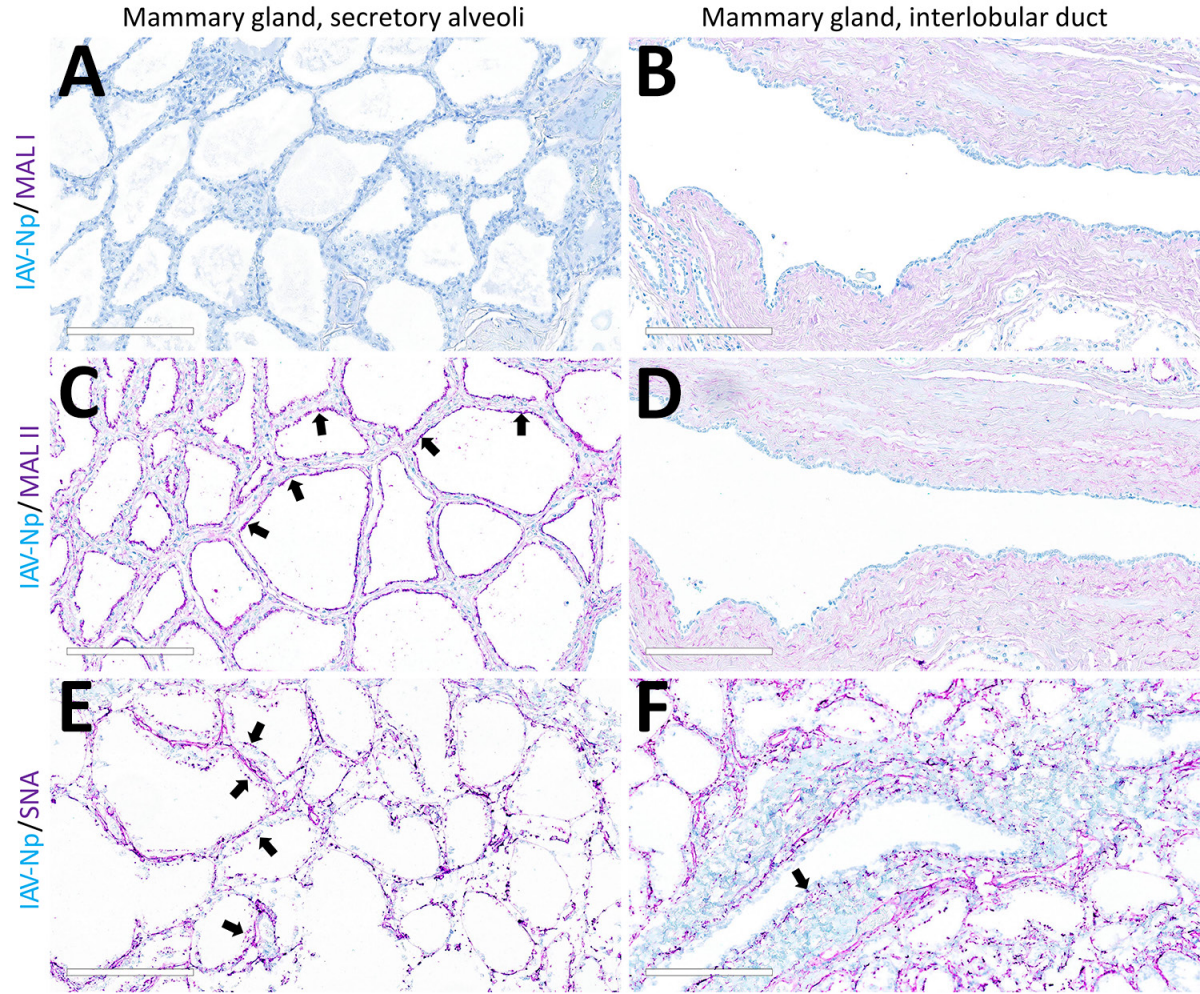
Unaffected region of the mammary gland from a US dairy cow infected with highly pathogenic avian influenza A(H5N1) virus, showing IAV-Np (teal chromogen), individually duplexed with MAL-I (magenta chromogen), MAL-II (magenta chromogen), and SNA (magenta chromogen) using chromogenic staining. Representative images of IAV-Np/MAL-I (A, B), IAV-Np/MAL-II (C, D), and IAV-Np/SNA (E, F) showing no IAV-Np labeling within unaffected mammary gland tissue sections. No MAL-I was detected in the mammary glandular (A) or interlobular duct epithelium (B). Within the alveolar gland epithelium, intense, granular, fibrillar labeling (arrows) of the apical portion labeling of MAL-II (C) was noted, with no epithelial labeling within the interlobular duct (D). Multifocal, strong, punctate, apical labeling (arrows) with SNA was observed within the mammary glandular epithelium (E). Scant apical labeling (arrow) was observed within the interlobular ductal epithelium with SNA (F). Scale bars indicate 200 μm. IAV-Np, influenza A virus nucleoprotein; MAL, *Maackia amurensis* lectin; SNA, *Sambucus nigra* lectin.

**Figure 5:**
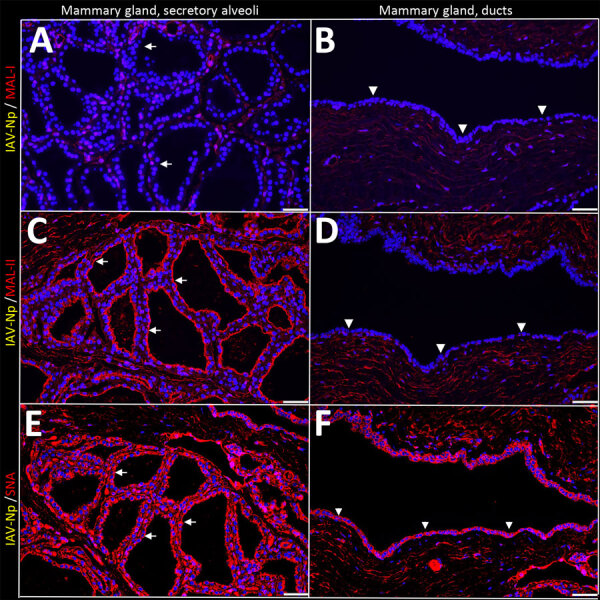
Unaffected region of the mammary gland from a US dairy cow infected with highly pathogenic avian influenza A(H5N1) virus, labeled with IAV-Np (yellow pseudocolor, DyLight 594), individually duplexed with MAL-I (red pseudocolor, Alexa Fluor 647), MAL-II (red pseudocolor, Alexa Fluor 647), and SNA (red pseudocolor, Alexa Fluor 647) using fluorescent staining. Representative merged images of IAV-Np/MAL-I (A, B), IAV-Np/MAL-II (C, D), and IAV-Np/SNA (E, F). IAV-Np labeling was not detected within unaffected mammary gland tissue sections. MAL-I labeling was neither observed in the epithelial cells (white arrows) lining the secretory alveoli (A) nor on the epithelial cells (white arrowheads) lining the interlobular ducts (B). Intense, apical, fibrillary MAL-II labeling was observed in the epithelial cells (white arrows) lining the secretory alveoli of unaffected mammary glands (C). MAL-II labeling was not observed in the epithelial cells (arrowheads) lining the interlobular ducts (D). Intense, apical, granular, membranous to cytoplasmic SNA labeling in the epithelial cells lining (white arrows) the secretory alveoli (E) and lining the interlobular ducts (arrowheads) (F) was observed. Scale bars indicate 50 μm. IAV-Np, influenza A virus nucleoprotein; MAL, *Maackia amurensis* lectin; SNA, *Sambucus nigra* lectin.

In the mammary gland, MAL-II (Figure 4; Figure 5, panels C, D) and SNA (Figure 4; Figure 5, panels E, F) labeling were expressed in unaffected secretory alveoli. We only observed unaffected interlobular ducts to have SNA labeling (Figure 4; Figure 5, panel F) but observed no detectable MAL-I (Figure 4; Figure 5, panels A, B) or IAV-Np labeling in unaffected mammary gland sections. SNA labeling was moderate to intense, granular to fibrillary, membranous to cytoplasmic, and predominately apically located within the alveolar lining epithelium (Figure 4; Figure 5, panel E), and the expression was abundant in the epithelial cells lining the interlobular ducts (Figure 4; Figure 5, panel F). MAL-II labeling was intense, fibrillary, membranous, and exclusively apical (Figure 4; Figure 5, panel C), but we did not not observe MAL-II labeling in interlobular duct epithelium (Figure 4; Figure 5, panel D).

Overall, lectin histochemistry results within unaffected (i.e., respiratory tract and mammary gland) and affected (i.e., mammary gland) tissues closely mirrored fluorescent microscopic findings (Table). Not surprisingly, the sensitivity and localization of labeling with chromogenic, lectin-based assays were not as definitive as fluorescent labeling. We detected no MAL-I labeling in the mammary gland with lectin histochemistry. 

**Figure 6:**
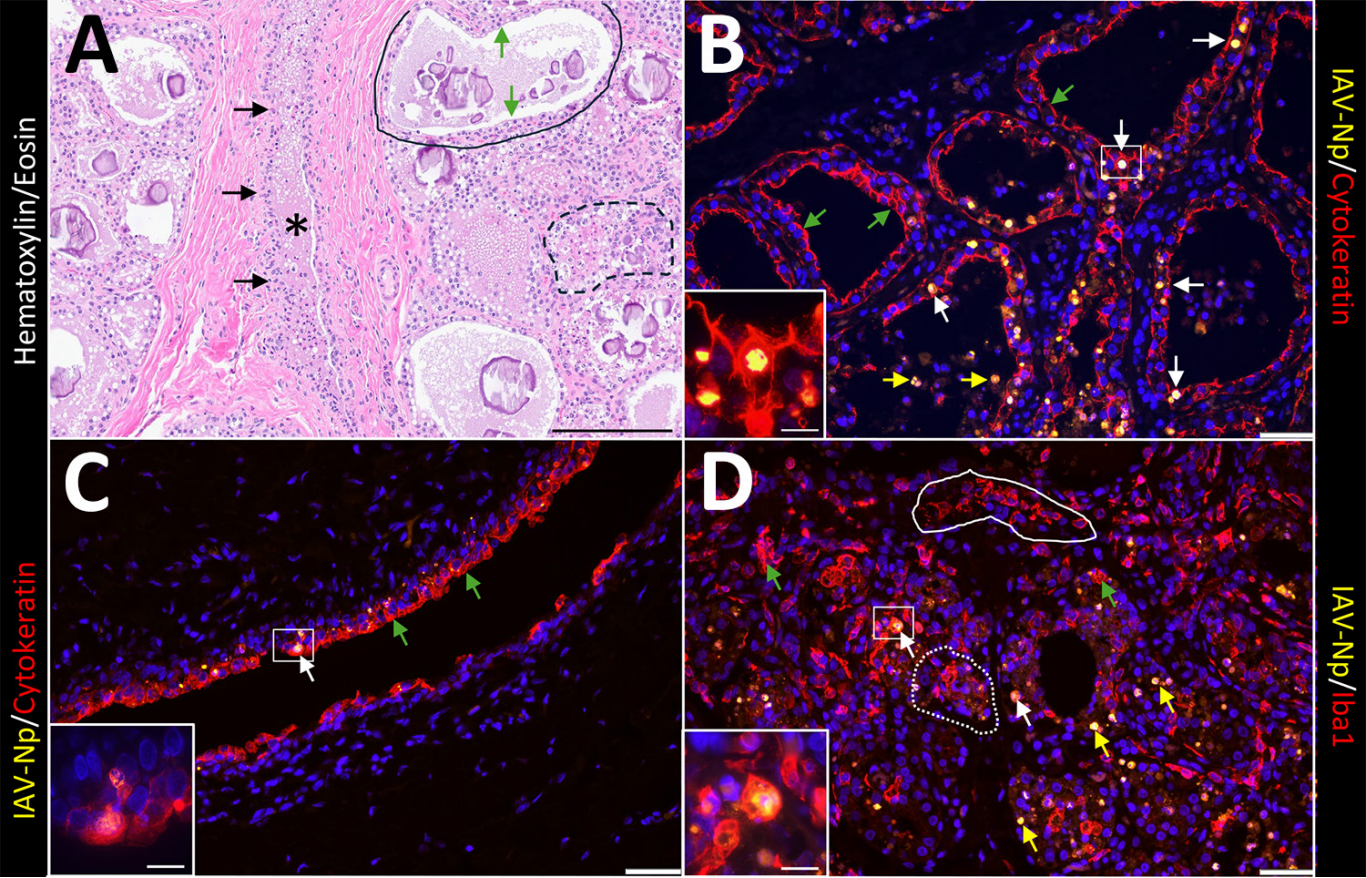
Infected region of the mammary gland from a US dairy cow infected with highly pathogenic avian influenza A(H5N1) virus, labeled with IAV-Np (yellow pseudocolor, DyLight 594), individually duplexed either with epithelial marker pan-cytokeratin (red pseudocolor, Alexa Fluor 647) (B, C) or macrophage or monocytic marker ionized calcium binding adaptor molecule 1 (Iba1) (red pseudocolor, Alexa Fluor 647) (D) using fluorescent labeling. A representative image from a hematoxylin and eosin stain section highlights the cellular architecture of the affected mammary gland (A). Unaffected secretory alveoli were observed in the section (solid black outline). Secretory alveoli variably contain eosinophilic proteinaceous material and corpora amylacea. The secretory alveoli were lined by cuboidal epithelial cells (green arrows) that were variably vacuolated. A few secretory alveoli were disrupted by inflammation (macrophages and neutrophils) and epithelial necrosis (dashed black outline). A single interlobular duct (black asterisk) lined by a bilayer of low cuboidal epithelial cells (black arrows) was observed. The duct contains eosinophilic proteinaceous fluids with scattered inflammatory cells (macrophages and neutrophils) and sloughed epithelium (A). Epithelial cells lining secretory alveoli (B) and interlobular ducts (C) were labeled with pancytokeratin as expected. IAV-Np intranuclear co-labeling (white arrows) in epithelial cells lining the secretory alveoli (B) and ducts (C). Intraluminal cells within secretory alveoli had intranuclear IAV-Np labeling (yellow arrows) that variably co-labeled with pan-cytokeratin. Likewise, Iba1 labeling (green arrows) was observed in the lumens of secretory alveoli or interlobular (alveolus labeling highlighted by white dotted outline) and interstitium (highlighted by the solid white outline) (D). Iba1 labeling was intense, diffuse, and cytoplasmic. IAV-Np intranuclear and intracytoplasmic labeling was less commonly co-labeled within Iba1 labeled cells (white arrows) (D). IAV-Np labeling was not observed in Iba1-labeled cells in the interstitium solid white outline). Insets highlight intranuclear labeling in panels C, B, and D, and the white boxes in the corresponding images represent the origin of the inset image. Scale bars indicate 200 μm (A), 50 μm (B, C, D), and 20 μm (insets). IAV-Np, influenza A virus nucleoprotein; Iba1, ionized calcium binding adaptor molecule 1.

**Figure 7:**
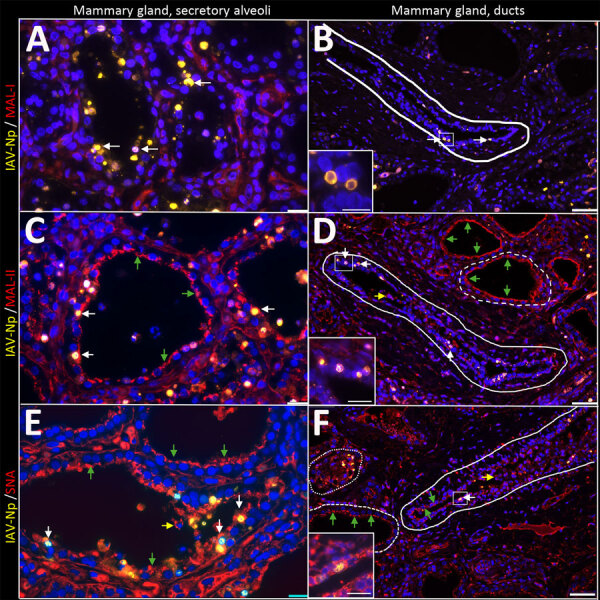
Infected region of the mammary gland from a US dairy cow infected with highly pathogenic avian influenza A(H5N1) virus, labeled with IAV-Np (yellow pseudocolor, DyLight 594), individually duplexed with MAL-I (red pseudocolor, Alexa Fluor 647), MAL-II (red pseudocolor, Alexa Fluor 647), and SNA (red pseudocolor, Alexa Fluor 647) using fluorescent staining. Representative merged images of IAV-Np/MAL-I (A, B), IAV-Np/MAL-II (C, D), and IAV-Np/SNA (E, F) are shown. Moderate to intense intranuclear and intracytoplasmic IAV-Np labeling (yellow, white arrows) was observed in epithelial cells lining the secretory alveolus of the mammary gland; however, no MAL-I labeling was detected (A). Rare, moderate intranuclear IAV-Np labeling (yellow, white arrows), but no MAL-I labeling was observed in epithelial cells lining the interlobular duct (solid white outline) (B). Intense, apical, fibrillary MAL-II labeling (green arrows) was observed in the epithelial cells lining the secretory alveoli of infected mammary glands (C). IAV-Np labeling was co-labeled with MAL-II in individual epithelial cells (white arrows). IAV-Np labeling (white arrows) was observed in epithelial cells of interlobular ducts (solid white outline) (D). MAL-II labeling was not observed in interlobular ducts but was detected in the unaffected secretory alveoli (dashed white outline) (D). Intraluminal cells with cytoplasmic MAL-II labeling were observed (yellow arrow) (D). Intense, apical, granular, membranous to cytoplasmic SNA labeling (green arrows) was observed in the epithelial cells lining the secretory alveoli of infected mammary glands (E). IAV-Np intranuclear labeling was co-labeled with SNA in individual epithelial cells (white arrows) in the secretory alveoli (E). SNA (green arrows) and IAV-Np (white arrows) were observed in epithelial cells of the interlobular ducts (dashed or solid white outline) and were co-labeled to individual ductal epithelial cells (F). Sloughed intraluminal cells had membranous SNA labeling (yellow arrows). Adjacent secretory alveoli had prominent SNA labeling (dashed white outline). The dotted white outline highlights a severely affected secretory gland (F). Insets highlight white boxed areas in panels B, D, and F. Scale bar indicate 20 μm (A, C, E), 50 μm (B, D, F), and 20 μm (insets). IAV-Np, influenza A virus nucleoprotein; MAL, *Maackia amurensis* lectin; SNA, *Sambucus nigra* lectin.

**Figure 8:**
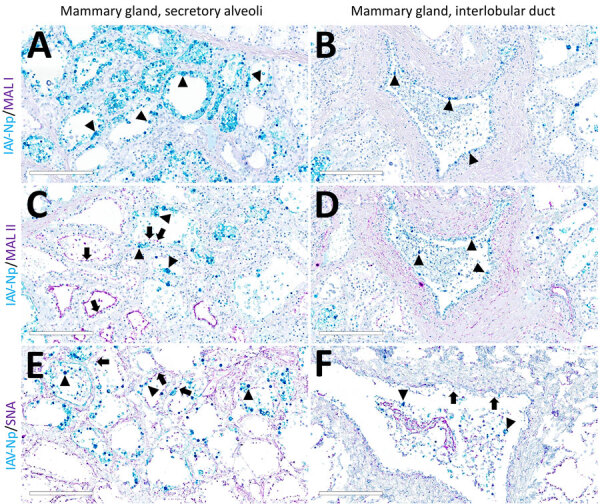
Infected region of the mammary gland from a US dairy cow infected with highly pathogenic avian influenza A(H5N1) virus, showing IAV-Np (teal chromogen), individually duplexed with MAL-I (magenta chromogen), MAL-II (magenta chromogen), and SNA (magenta chromogen) using chromogenic staining. Representative images IAV-Np/MAL-I (A, B), IAV-Np/MAL-II (C, D), and IAV-Np/SNA (E, F) in infected mammary gland and interlobular duct are shown. Strong intracytoplasmic and intranuclear immunoreactivity with IAV-Np was observed within the glandular epithelium of the mammary alveolus and interlobular ducts (arrowheads) with no substantial MAL-I labeling (A, B). Scant, multifocal, apical, punctate MAL-II labeling was observed in degenerative mammary gland epithelial cells (arrows) with strong, intranuclear, and intracytoplasmic immunoreactivity for IAV-Np (arrowheads) (C). Interlobular ductal epithelium exhibits strong, multifocal intranuclear and intracytoplasmic immunoreactivity to IAV-Np in attenuated and sloughed cells (arrowheads) with no substantial epithelial MAL-II detected (D). Strong, multifocal, punctate, apical labeling with SNA in attenuated or degenerative epithelial cells (arrows) with strong, intranuclear, and intracytoplasmic immunoreactivity for IAV-Np (arrows) was observed within mammary gland epithelium (E). Scant, multifocal, delicate, apical labeling for SNA (arrows) within ductal epithelium with strong, intranuclear, and intracytoplasmic immunoreactivity within sloughed, intraluminal epithelial cells for IAV-Np (arrowheads) was observed in an interlobular duct (F). Scale bars indicate 200 μm. IAV-Np, influenza A virus nucleoprotein; MAL, *Maackia amurensis* lectin; SNA, *Sambucus nigra* lectin.

As described previously, the HPAI H5N1 virus–infected mammary gland had multifocal acute moderate mastitis with prominent epithelial changes in the secretory alveoli and ducts with sloughed intraluminal epithelial cells, macrophages, and neutrophils (Figure 6, panel A). We used pancytokeratin (epithelial marker) and Iba1 (macrophage marker) to evaluate the intracellular distribution of IAV-Np. We observed co-labeling of IAV-Np labeling with pan-cytokeratin in cells lining the secretory alveoli and interlobular ducts (Figure 6, panels B, C). IAV-Np labeling was more widely distributed and intense in the secretory alveolar epithelium than in the ductal epithelium. Some intraluminal cells within secretory alveoli and ducts had both intranuclear and cytoplasmic IAV-Np expression. Intraluminal cells are commonly labeled with pancytokeratin (sloughed epithelial cells) and rare Iba1-positive cells (macrophages) (Figure 6, panels B, D). Interstitial Iba1-labeled cells did not have IAV-Np co-labeling (Figure 6, panel D). We observed co-labeling with intranuclear IAV-Np labeling and MAL-II (Figure 7; Figure 8, panel C) and SNA (Figure 7; Figure 8, panel E) in epithelial cells lining the secretory alveoli. We observed only co-labeling with intranuclear IAV-Np labeling and SNA in the ductal epithelial cells (Figure 7; Figure 8, panel F), given that we did not observe MAL-II labeling in the ducts (Figure 7; Figure 8, panel D). MAL-I labeling could not be detected with IAV-Np because MAL-I labeling was not observed in the secretory alveoli or ducts (Figure 7; Figure 8, panels A, B).

## Discussion

The presence of HPAI H5N1 virus in dairy cattle, more so in the mammary gland and milk, once again highlights the importance of IAV adaptability to other nontraditional species and cross-species transmission. This finding reiterates the need for active IAV surveillance efforts in animal species. Like coronaviruses, IAVs have a broad host range involving avian and mammal species. RNA viruses are inherently error-prone, and infection of new host species gives the virus additional opportunities to replicate and subsequently mutate to be better adapt to novel hosts. Another contributing factor to the broad host range of IAV is the presence of virus SA receptors. Many RNA and DNA viruses, including SARS-CoV-2 (co-receptor), use SA as host receptors for initial attachment and entry into the cells (*18*).

In general, IAVs originating from humans and swine preferentially bind to SA linked to galactose through α2,6 linkage (*14*,*19*), whereas the IAVs of avian and equine species preferentially bind to SA linked to galactose through α2,3 linkage (*10*,*20*,*21*). The susceptibility of a host to IAV infection is determined by the type of SA receptor present on the host cell surface, along with other host factors. Previous studies indicated that the HPAI H5N1 virus preferentially binds to α2,3-linked SA receptors (*22*–*24*) and is expressed abundantly in avian upper airways and their gastrointestinal tracts (*20*,*21*). IAV strains established in mammals, particularly in humans and swine, exhibit a higher tropism or affinity for α2,6-linked SA receptors, which are predominantly expressed on the epithelial lining of their upper airways (*23*,*25*).

SAs are 9-carbon carboxylated monosaccharides synthesized in animals but not in plants (*26*,*27*). The most common forms of SAs are N-acetylneuraminic acid (Neu5Ac) or N-glycoloylneuraminic acid (Neu5Gc) (*26*). Although IAVs bind to both Neu5Ac and Neu5Gc, the role of Neu5Gc was reported to be nonfunctional (*28*,*29*). SAs are also a major component of milk (*30*). Cattle and goat milk predominantly contain Neu5Gc versus Neu5Ac, and their content decreases with the progression of the lactation stage (*31*,*32*).

Our study explores the expression and distribution of SAs in the respiratory tract and mammary glands of Holstein dairy cows naturally infected with HPAI H5N1 virus. The lectins such as SNA, MAL-I, and MAL-II used in this study specifically detect the Neu5Ac form of SA (*12*,*33*). Our findings suggest that SAs are widely expressed in these tissues with predominant SA α2,3-gal-β (1–3) GalNAc (MAL-II), followed by α2,6-linked SA (SNA) and SAα2,3-gal-β (1–4) GlcNAc (MAL-I). However, the expression and distribution varied across tissues. The lectin binding specificity was further validated in a normal mammary gland of a biobanked slide, in which, after removing SAs (90%) by using sialidase A, lectin expression was negative, and so was low pathogenicity H5 virus binding affinity in those tissues (Appendix Figures 2–4). Although HPAI H5N1 and low pathogenicity avian influenza H5N9 viruses were of completely different clades, the findings suggest that no limitations would exist regarding receptor availability and distribution in the bovine mammary gland for IAVs to bind.

In the respiratory tract, the dairy cows expressed both SAα2,3-gal and SAα2,6-gal. Unlike in pigs and humans (*17*,*19*), the mammal-specific SAα2,6-gal in cattle is mainly confined to the subepithelial region of the trachea, occasionally in the goblet cells and subepithelial glands. The expression of SAα2,3-gal continued on the epithelium of bronchi to alveolar pneumocytes. The findings suggest that avian IAVs have the potential binding affinity to the bovine respiratory tract. In the mammary gland, both SA α2,3-gal-β (1–3) GalNAc (MAL-II) and SAα2,6-gal (SNA) were expressed, and co-localization was mainly observed in the alveolar gland and intralobular duct epithelium, whereas SA α2,3-gal-β (1–4) GlcNAc was minimal and confined to interstitial regions. Mammalian- and avian-origin IAVs might bind to cells in the mammary gland. Our findings were further supported by the co-localization of intranuclear and cytoplasmic IAV-Np of HPAI H5N1 virus in some intraluminal cells within secretory alveoli and ducts, suggesting possible virus replication.

The exact pathogenesis of mastitis caused by HPAI H5N1 virus in dairy cattle remains to be elucidated. The initial evaluation of IAV mastitis in dairy cattle identified strong epitheliotropism on the basis of on histologic, immunohistochemical, and FA evaluation. The intramacrophagic viral IAV-Np could be the result of phagocytosis or active infection with virus replication. Intranuclear localization observed in this case may suggest viral replication in macrophages. Previous studies have shown that H5N1 virus replicates efficiently in human macrophages (*34*). Even in pigs, pulmonary alveolar macrophages expressing SA receptors have been shown to be susceptible to IAVs and undergo rapid apoptosis (*35*). Phagocytosis of viral antigen also is probably occurring given that only intraluminal macrophages mixed with inflammatory exudate contained viral antigen, whereas the interstitial macrophages did not. Overall, resident or infiltrating macrophages in the bovine mammary gland may be susceptible to infection, but further studies are required.

IAV infections are typically respiratory tract infections. Severe infections can result in a systemic inflammatory response, but the spread of IAV to tissues outside of the respiratory tract is rare (*36*). HPAI H5N1 and H5N8 viruses been reported to cause lesions outside of the respiratory tract, including encephalitis and myocarditis, in humans and wild mammals (*37*–*39*). The multifocal random pattern of viral distribution and lesion development in dairy cattle may suggest either a hematogenous spread (viremia or lymphohistiocytic trafficking) or ascending infection from the teat sinus. Although it is beyond the scope of this article, it will be interesting to explore other suspected routes of infection. The involvement of other organ systems (e.g., the gastrointestinal tract through pancreatic tropism), have been observed in naturally infected poultry and cats with systemic disease (*38*,*40*,*41*).

Virus attachment through hemagglutinin to host cell SA and the association between the attachment pattern, disease pathogenesis, and transmission efficiency is complex. Slight changes in the receptor-binding activity, as occurs with changes in pH, can influence the functional balance of the IAV infection process (*42*–*44*). The pH required for the fusion of many avian-adapted IAVs is less acidic than human IAVs (*42*,*44*). The pH of normal cow milk is mildly acidic (6.3–6.9), and the pH of mastitis milk often increases (*45*); however, pH was not measured in the submitted milk sample. It is possible that the mildly acidic environment in the bovine mammary gland, coupled with the presence of SA receptors across the lacteal ducts, can be some of the predisposing factors for HPAI H5N1 virus infection in dairy cattle. As hypothesized earlier, imbalances in pH-associated hemagglutinin and neuraminidase conformational changes may lead to inefficient viral progeny release (*42*). However, those observations require additional studies, and the pH of milk from experimentally infected cows should be assessed temporally to assess how these imbalances may affect viral replication.

Sporadic human cases of H5N1 virus infection have occurred when humans are in close and prolonged contact with birds (*46*,*47*). Humans have some α2,3-linked SA receptors deep within their lungs (*14*), and prolonged close contact with infected birds is postulated to cause infection attributable to inhaling large amounts of virus from those birds with introduction into the deeper recesses of the lungs (*48*,*49*). Even in the current HPAI H5N1 outbreak, there was a reported case of a dairy farm worker who had direct and close exposure to dairy cows that had onset of bilateral conjunctivitis and was later confirmed positive for H5N1 clade 2.3.4.4b virus through rRT-PCR and sequence analysis (*50*). Sequence analysis of the hemagglutinin gene in HPAI H5N1 virus samples from cattle and humans reportedly lacked changes in the receptor-binding affinity (i.e., the virus still preferentially binds to SA α2–3–linked receptors) (*50*). However, the sample size tested in both dairy cattle (2 cows) and humans (1 human) is relatively small. Continued sequence analysis of identified cases is important to assess viral mutations and potential adaptation to other species. This monitoring will assist preparedness and decision making.

From a public health standpoint, there is an urgent need to understand why these avian influenza viruses are now infecting so many mammal species. There is also a need to understand why and how often these viruses infect humans. Our study is an initial foray into answering those questions. Ongoing surveillance and sequence comparisons of HPAI viruses in various mammals are needed to better understand spillover events and the underlying pathogenic mechanisms. A better understanding of viral host range will help inform public health decisions and guide research to help prevent future influenza epidemics.

Appendix Additional information about sialic acid receptor specificity in mammary gland of dairy cattle infected with highly pathogenic avian influenza A(H5N1) virus.

## References

[1] Burrough ER, Magstadt DR, Petersen B, Timmermans SJ, Gauger PC, Zhang J, et al. Highly pathogenic avian influenza A(H5N1) clade 2.3.4.4b virus infection in domestic dairy cattle and cats, United States, 2024. Emerg Infect Dis. 2024;30:30. 10.3201/eid3007.240508 38683888 PMC11210653

[2] US Department of Agriculture, Animal and Plant Health Inspection Service. Federal and state veterinary, public health agencies share update on HPAI detection in Kansas, Texas dairy herds. 2024 [cited 2024 May 5]. https://www.aphis.usda.gov/news/agency-announcements/federal-state-veterinary-public-health-agencies-share-update-hpai

[3] Gilbertson B, Subbarao K. Mammalian infections with highly pathogenic avian influenza viruses renew concerns of pandemic potential. J Exp Med. 2023;220:e20230447. 10.1084/jem.20230447 37326966 PMC10276204

[4] US Department of Agriculture, US Department of Health and Human Services. USDA, HHS announce new actions to reduce impact and spread of H5N1. 2024 [cited 2024 May 5]. https://www.usda.gov/media/press-releases/2024/05/10/usda-hhs-announce-new-actions-reduce-impact-and-spread-h5n1

[5] Bevins SN, Shriner SA, Cumbee JC Jr, Dilione KE, Douglass KE, Ellis JW, et al. Intercontinental movement of highly pathogenic avian influenza A(H5N1) clade 2.3.4.4 virus to the United States, 2021. Emerg Infect Dis. 2022;28:1006–11. 10.3201/eid2805.220318 35302933 PMC9045435

[6] Caliendo V, Lewis NS, Pohlmann A, Baillie SR, Banyard AC, Beer M, et al. Transatlantic spread of highly pathogenic avian influenza H5N1 by wild birds from Europe to North America in 2021. Sci Rep. 2022;12:11729. 10.1038/s41598-022-13447-z 35821511 PMC9276711

[7] Centers for Disease Control and Prevention. Emergence and evolution of H5N1 bird flu. 2024 [cited 2024 May 5]. https://www.cdc.gov/flu/avianflu/communication-resources/bird-flu-origin-infographic.html

[8] Elsmo EJ, Wünschmann A, Beckmen KB, Broughton-Neiswanger LE, Buckles EL, Ellis J, et al. Highly pathogenic avian influenza A(H5N1) virus clade 2.3.4.4b infections in wild terrestrial mammals, United States, 2022. Emerg Infect Dis. 2023;29:2451–60. 10.3201/eid2912.230464 37987580 PMC10683806

[9] US Department of Agriculture, Animal and Plant Health Inspection Service, Wildlife Services. National Wildlife Disease Program: HPAI detections in mammals. 2024 [cited 2024 May 13]. https://www.aphis.usda.gov/livestock-poultry-disease/avian/avian-influenza/hpai-detections/mammals

[10] Suzuki Y, Ito T, Suzuki T, Holland RE Jr, Chambers TM, Kiso M, et al. Sialic acid species as a determinant of the host range of influenza A viruses. J Virol. 2000;74:11825–31. 10.1128/JVI.74.24.11825-11831.2000 11090182 PMC112465

[11] Arruda B, Baker ALV, Buckley A, Anderson TK, Torchetti M, Bergeson NH, et al. Divergent pathogenesis and transmission of highly pathogenic avian influenza A(H5N1) in swine. Emerg Infect Dis. 2024;30:738–51. 10.3201/eid3004.231141 38478379 PMC10977838

[12] Nicholls JM, Chan RWY, Russell RJ, Air GM, Peiris JSM. Evolving complexities of influenza virus and its receptors. Trends Microbiol. 2008;16:149–57. 10.1016/j.tim.2008.01.008 18375125

[13] Venkatesh D, Anderson TK, Kimble JB, Chang J, Lopes S, Souza CK, et al. Antigenic characterization and pandemic risk assessment of North American H1 influenza a viruses circulating in swine. Microbiol Spectr. 2022;10:e0178122. 10.1128/spectrum.01781-22 36318009 PMC9769642

[14] Shinya K, Ebina M, Yamada S, Ono M, Kasai N, Kawaoka Y. Avian flu: influenza virus receptors in the human airway. Nature. 2006;440:435–6. 10.1038/440435a 16554799

[15] Angata T, Varki A. Chemical diversity in the sialic acids and related alpha-keto acids: an evolutionary perspective. Chem Rev. 2002;102:439–69. 10.1021/cr000407m 11841250

[16] Lin SJH, Helm ET, Gabler NK, Burrough ER. Acute infection with *Brachyspira hyodysenteriae* affects mucin expression, glycosylation, and fecal MUC5AC. Front Cell Infect Microbiol. 2023;12:1042815. 10.3389/fcimb.2022.1042815 36683692 PMC9852840

[17] Nelli RK, Kuchipudi SV, White GA, Perez BB, Dunham SP, Chang KC. Comparative distribution of human and avian type sialic acid influenza receptors in the pig. BMC Vet Res. 2010;6:4. 10.1186/1746-6148-6-4 20105300 PMC2832630

[18] Matrosovich M, Herrler G, Klenk HD. Sialic acid receptors of viruses. In: Gerardy-Schahn R, Delannoy P, Von Itzstein M, editors. SialoGlyco chemistry and biology II. Cham (Switzerland): Springer International Publishing; 2013. p. 1–28 [cited 2024 May 5]. http://link.springer.com/10.1007/128_2013_466

[19] van Riel D, Munster VJ, de Wit E, Rimmelzwaan GF, Fouchier RA, Osterhaus AD, et al. Human and avian influenza viruses target different cells in the lower respiratory tract of humans and other mammals. Am J Pathol. 2007;171:1215–23. 10.2353/ajpath.2007.070248 17717141 PMC1988871

[20] Kuchipudi SV, Nelli R, White GA, Bain M, Chang KC, Dunham S. Differences in influenza virus receptors in chickens and ducks: Implications for interspecies transmission. J Mol Genet Med. 2009;3:143–51. 10.4172/1747-0862.1000026 19565022 PMC2702077

[21] Costa T, Chaves AJ, Valle R, Darji A, van Riel D, Kuiken T, et al. Distribution patterns of influenza virus receptors and viral attachment patterns in the respiratory and intestinal tracts of seven avian species. Vet Res (Faisalabad). 2012;43:28. 10.1186/1297-9716-43-28 22489675 PMC3368784

[22] Kandeil A, Patton C, Jones JC, Jeevan T, Harrington WN, Trifkovic S, et al. Rapid evolution of A(H5N1) influenza viruses after intercontinental spread to North America. Nat Commun. 2023;14:3082. 10.1038/s41467-023-38415-7 37248261 PMC10227026

[23] Centers for Disease Control and Prevention. Technical report: highly pathogenic avian influenza A(H5N1) viruses. 2024 [cited 2024 May 5]. https://www.cdc.gov/flu/avianflu/spotlights/2023-2024/h5n1-technical-report_april-2024.htm

[24] van Riel D, Munster VJ, de Wit E, Rimmelzwaan GF, Fouchier RA, Osterhaus AD, et al. H5N1 virus attachment to lower respiratory tract. Science. 2006;312:399. 10.1126/science.1125548 16556800

[25] Gambaryan A, Yamnikova S, Lvov D, Tuzikov A, Chinarev A, Pazynina G, et al. Receptor specificity of influenza viruses from birds and mammals: new data on involvement of the inner fragments of the carbohydrate chain. Virology. 2005;334:276–83. 10.1016/j.virol.2005.02.003 15780877

[26] Ghosh S. Sialic acid and biology of life: an introduction. In: sialic acids and sialoglycoconjugates in the biology of life, health and disease. Amsterdam: Elsevier; 2020. p. 1–61 [cited 2024 May 5]. https://linkinghub.elsevier.com/retrieve/pii/B9780128161265000019

[27] Varki A, Schnaar RL, Schauer R. Sialic acids and other nonulosonic acids. In: Varki A, Cummings RD, Esko JD, Stanley P, Hart GW, Aebi M, et al., editors. Essentials of glycobiology. 3rd edition. Cold Spring Harbor (NY): Cold Spring Harbor Laboratory Press; 2015 [cited 2024 May 5]. https://www.ncbi.nlm.nih.gov/books/NBK453082 28876847

[28] Kuchipudi SV, Nelli RK, Gontu A, Satyakumar R, Surendran Nair M, Subbiah M. Sialic acid receptors: the key to solving the enigma of zoonotic virus spillover. Viruses. 2021;13:262. 10.3390/v13020262 33567791 PMC7915228

[29] Spruit CM, Nemanichvili N, Okamatsu M, Takematsu H, Boons GJ, de Vries RP. N-glycolylneuraminic acid in animal models for human influenza A virus. Viruses. 2021;13:815. 10.3390/v13050815 34062844 PMC8147317

[30] Sharma R, Ahlawat S, Sharma H, Aggarwal RAK, Sharma V, Tantia MS. Variable sialic acid content in milk of Indian cattle and buffalo across different stages of lactation. J Dairy Res. 2019;86:98–101. 10.1017/S002202991800081X 30520408

[31] De Sousa YRF, Da Silva Vasconcelos MA, Costa RG, De Azevedo Filho CA, De Paiva EP, Queiroga RDCRDE. Sialic acid content of goat milk during lactation. Livest Sci. 2015;177:175–80. 10.1016/j.livsci.2015.04.005

[32] Puente R, Hueso P. Lactational changes in the N-glycoloylneuraminic acid content of bovine milk gangliosides. Biol Chem Hoppe Seyler. 1993;374:475–8. 10.1515/bchm3.1993.374.7-12.475 8216898

[33] Sata T, Roth J, Zuber C, Stamm B, Heitz PU. Expression of α 2,6-linked sialic acid residues in neoplastic but not in normal human colonic mucosa. A lectin-gold cytochemical study with *Sambucus nigra* and *Maackia amurensis* lectins. Am J Pathol. 1991;139:1435–48. 1661075 PMC1886452

[34] Westenius V, Mäkelä SM, Julkunen I, Österlund P. Highly pathogenic H5N1 influenza A virus spreads efficiently in human primary monocyte-derived macrophages and dendritic cells. Front Immunol. 2018;9:1664. 10.3389/fimmu.2018.01664 30065728 PMC6056608

[35] Chang P, Kuchipudi SV, Mellits KH, Sebastian S, James J, Liu J, et al. Early apoptosis of porcine alveolar macrophages limits avian influenza virus replication and pro-inflammatory dysregulation. Sci Rep. 2015;5:17999. 10.1038/srep17999 26642934 PMC4672291

[36] Taubenberger JK, Morens DM. The pathology of influenza virus infections. Annu Rev Pathol. 2008;3:499–522. 10.1146/annurev.pathmechdis.3.121806.154316 18039138 PMC2504709

[37] Zhang Z, Zhang J, Huang K, Li KS, Yuen KY, Guan Y, et al. Systemic infection of avian influenza A virus H5N1 subtype in humans. Hum Pathol. 2009;40:735–9. 10.1016/j.humpath.2008.08.015 19121843 PMC7112124

[38] Reperant LA, van Amerongen G, van de Bildt MWG, Rimmelzwaan GF, Dobson AP, Osterhaus ADME, et al. Highly pathogenic avian influenza virus (H5N1) infection in red foxes fed infected bird carcasses. Emerg Infect Dis. 2008;14:1835–41. 10.3201/eid1412.080470 19046504 PMC2634621

[39] Floyd T, Banyard AC, Lean FZX, Byrne AMP, Fullick E, Whittard E, et al. Encephalitis and death in wild mammals at a rehabilitation center after infection with highly pathogenic avian influenza A(H5N8) virus, United Kingdom. Emerg Infect Dis. 2021;27:2856–63. 10.3201/eid2711.211225 34670647 PMC8544989

[40] Sillman SJ, Drozd M, Loy D, Harris SP. Naturally occurring highly pathogenic avian influenza virus H5N1 clade 2.3.4.4b infection in three domestic cats in North America during 2023. J Comp Pathol. 2023;205:17–23. 10.1016/j.jcpa.2023.07.001 37586267

[41] Arruda PHE, Stevenson GW, Killian ML, Burrough ER, Gauger PC, Harmon KM, et al. Outbreak of H5N2 highly pathogenic avian Influenza A virus infection in two commercial layer facilities: lesions and viral antigen distribution. J Vet Diagn Invest. 2016;28:568–73. 10.1177/1040638716658929 27423731

[42] Wagner R, Matrosovich M, Klenk HD. Functional balance between haemagglutinin and neuraminidase in influenza virus infections. Rev Med Virol. 2002;12:159–66. 10.1002/rmv.352 11987141

[43] Short KR, Richard M, Verhagen JH, van Riel D, Schrauwen EJA, van den Brand JMA, et al. One health, multiple challenges: The inter-species transmission of influenza A virus. One Health. 2015;1:1–13. 10.1016/j.onehlt.2015.03.001 26309905 PMC4542011

[44] Galloway SE, Reed ML, Russell CJ, Steinhauer DA. Influenza HA subtypes demonstrate divergent phenotypes for cleavage activation and pH of fusion: implications for host range and adaptation. PLoS Pathog. 2013;9:e1003151. 10.1371/journal.ppat.1003151 23459660 PMC3573126

[45] Kandeel SA, Megahed AA, Ebeid MH, Constable PD. Ability of milk pH to predict subclinical mastitis and intramammary infection in quarters from lactating dairy cattle. J Dairy Sci. 2019;102:1417–27. 10.3168/jds.2018-14993 30343916

[46] Van Kerkhove MD, Mumford E, Mounts AW, Bresee J, Ly S, Bridges CB, et al. Highly pathogenic avian influenza (H5N1): pathways of exposure at the animal-human interface, a systematic review. PLoS One. 2011;6:e14582. 10.1371/journal.pone.0014582 21283678 PMC3025925

[47] Bertran K, Balzli C, Kwon YK, Tumpey TM, Clark A, Swayne DE. Airborne transmission of highly pathogenic influenza virus during processing of infected poultry. Emerg Infect Dis. 2017;23:1806–14. 10.3201/eid2311.170672 29047426 PMC5652435

[48] Kumlin U, Olofsson S, Dimock K, Arnberg N. Sialic acid tissue distribution and influenza virus tropism. Influenza Other Respir Viruses. 2008;2:147–54. 10.1111/j.1750-2659.2008.00051.x 19453419 PMC4941897

[49] Maginnis MS. Virus–receptor interactions: the key to cellular invasion. J Mol Biol. 2018;430:2590–611. 10.1016/j.jmb.2018.06.024 29924965 PMC6083867

[50] Uyeki TM, Milton S, Abdul Hamid C, Reinoso Webb C, Presley SM, Shetty V, et al. Highly pathogenic avian influenza A(H5N1) virus infection in a dairy farm worker. N Engl J Med. [Epub ahead of print]. N Engl J Med. 2024;•••:c2405371. 10.1056/NEJMc2405371 38700506

