# Supplementary material for: Sialic Acid Receptor Specificity in Mammary Gland of Dairy Cattle Infected with Highly Pathogenic Avian Influenza A(H5N1) Virus
Source: Emerg Infect Dis. 2024 Jul;30(7):1361–73. doi: 10.3201/eid3007.240689 (PMC11210646; doi:10.3201/eid3007.240689)
Supplement: Appendix — Additional information about sialic acid receptor specificity in mammary gland of dairy cattle infected with highly pathogenic avian influenza A(H5N1) virus. [file 24-0689-Techapp-s1.pdf]

Article DOI: https://doi.org/10.3201/eid3007.240689

EID cannot ensure accessibility for supplementary materials supplied by authors. Readers who have difficulty accessing supplementary content should contact the authors for assistance.

Sialic Acid Receptor Specificity in Mammary Gland of Dairy Cattle Infected with Highly Pathogenic Avian Influenza A(H5N1) Virus

Appendix

Viral Binding and Sialidase Assays

Bovine mammary glands were used for virus binding assay with heat-inactivated low pathogenic avian influenza (LPAI) H5N9 influenza A virus (Avian Influenza A/Emu/NY/12716–3/1994). A previously described (*1*) protocol was modified for dual staining with lectins and virus binding, using pre-treated slides with/without sialidase-A. In brief, deparaffinized and citrate buffer retrieved tissue sections were pre-treated with 0.4 U/mL Sialidase-A (Agilent Technologies) in 50 mM sodium phosphate buffer (pH 6.0) or buffer alone for 24 h at 37°C. The sections were washed three times with TBST and inoculated with 2 μg/ml TPCK trypsin-treated H5N9 influenza A virus for another 24 h at 37°C. After removing the virus and washing three times with TBST, the sections were ready for staining procedures. Tissue sections were treated with Animal-Free Blocker R.T.U (Vector Laboratories) for 30 min at room temperature and followed by lectin staining procedure as described above. The sections were washed three times with TBST and incubated with primary antibody for mouse monoclonal antibody for anti-influenza A H5 (hemagglutinin 5) antigen (Bio-Rad Laboratories) for 2 h at room temperature. For secondary antibodies, all SNA and MAL-I stained sections (i.e., Fluorescein labeled) were incubated with Goat anti-Mouse IgG (H+L) cross-adsorbed secondary antibody conjugated to Alexa Fluor-568 and MAL-II labeled sections with AffiniPure Donkey Anti-Mouse IgG (H+L) conjugated Alexa Fluor-488 secondary antibody for 2 h at room temperature. Finally, the sections were washed three times with TBST, air-dried, and mounted with ProLong Diamond Antifade Mountant with DAPI (Thermo Fisher Scientific). Healthy mammary glands from an

unrelated Holstein dairy cattle were used as controls to perform sialidase and virus binding assays.

Reference

1. Nelli RK, Kuchipudi SV, White GA, Perez BB, Dunham SP, Chang KC. Comparative distribution of human and avian type sialic acid influenza receptors in the pig. BMC Vet Res. 2010;6:4. PubMed https://doi.org/10.1186/1746-6148-6-4

Appendix Table. Lectin histochemistry and immunohistochemistry reagents*

Staining method	Conjugate	Working concentration	Manufacturer	Product number
Lectin staining				
Maackia amurensis -I	Biotinylated	20 µg/mL	Vector Laboratories	B-1315–2
Maackia amurensis -II	Biotinylated	4 µg/mL	Vector Laboratories	B-1265–1
Sambucus nigra	Biotinylated	20 µg/mL	Vector Laboratories	B-1305–2
Streptavidin	Alexa Fluor-647	2 µg/mL	ThermoFisher Scientific	S32357
Influenza A virus nucleoprotein staining				
Rabbit monoclonal anti-IAV-Np	Alexa Fluor-594	2 µg/mL	Novus Biologicals	NBP3–12741DL594
Mouse monoclonal anti-IAV-Np (hybridoma suspension - H16-L10–4R5)	None	1:100	ATCC	HB-65
Immunofluorescent staining for epithelial cells and macrophages				
Mouse monoclonal anti-human cytokeratin, Clone AE1/AE3	None	2 µg/mL	Agilent Technologies	GA053
Rabbit monoclonal anti-Iba1	None	1 µg/mL	Abcam	EPR16588
Universal anti-rabbit/mouse detection kit	None	Manufacturer specifications	Agilent Technologies	K067589–2
Virus binding staining				
Mouse monoclonal anti-influenza A H5	None	20 µg/mL	Bio-Rad Laboratories	MCA2661
Goat anti-mouse IgG (H+L) cross-adsorbed secondary antibody	Alexa Fluor-568	4 µg/mL	ThermoFisher Scientific	A-11004
Donkey anti-mouse IgG (H+L) secondary antibody	Alexa Fluor-488	1 µg/mL	Jackson ImmunoResearch	715–545–150

*Iba1, ionizing binding calciun adaptor protein-1; IAV-Np, influenza virus nucleoprotien; H5, hemagluttin 5.

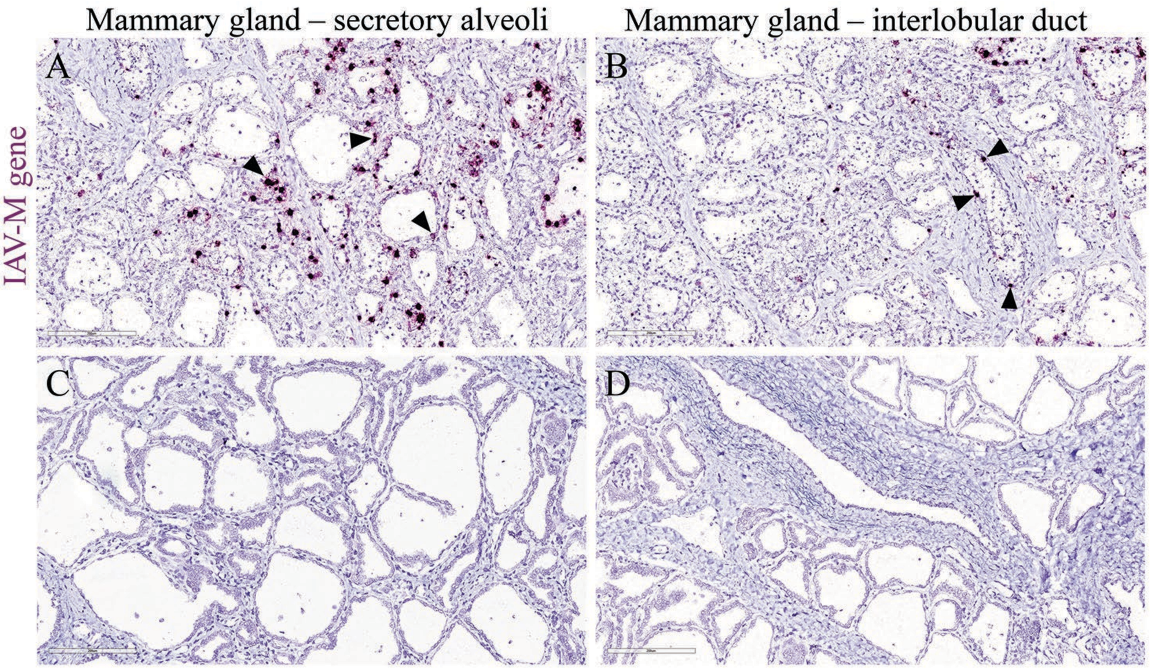

Appendix Figure 1. RNAscope in situ hybridization assay image of affected regions of the mammary gland from a dairy cow with HPAI-H5N1 showing detection of IAV-M gene nucleic acid (magenta). The affected portion of the mammary gland has strong, intranuclear and perinuclear labeling (arrows) (A) within the epithelium lining glands. The interlobular duct epithelium within affected tissue sections has sporadic, intranuclear and cytoplasmic labeling (arrows) (B). No labeling was detected within unaffected portions of the mammary gland or interlobular ducts (C, D). All images were original magnification x 200; Scale bar = 200 µm.

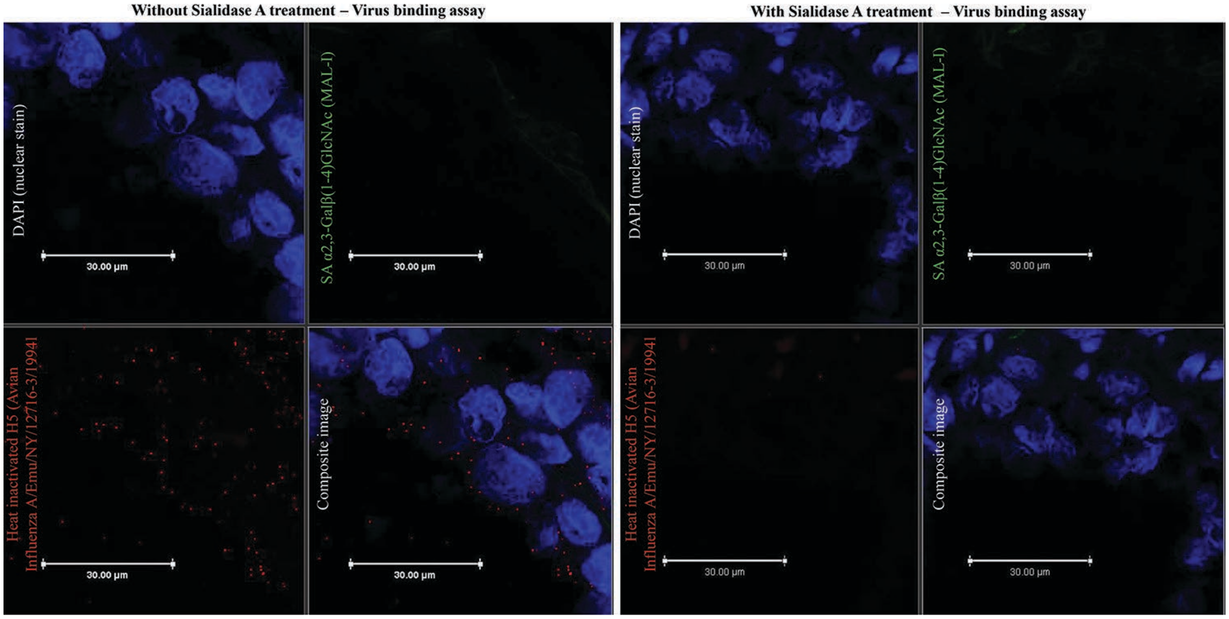

Appendix Figure 2. MAL-I lectin (green) and IAV-H5 (red) virus binding assays on normal healthy bovine mammary gland treated with/without sialidase-A. Scale bar = 30 µm.

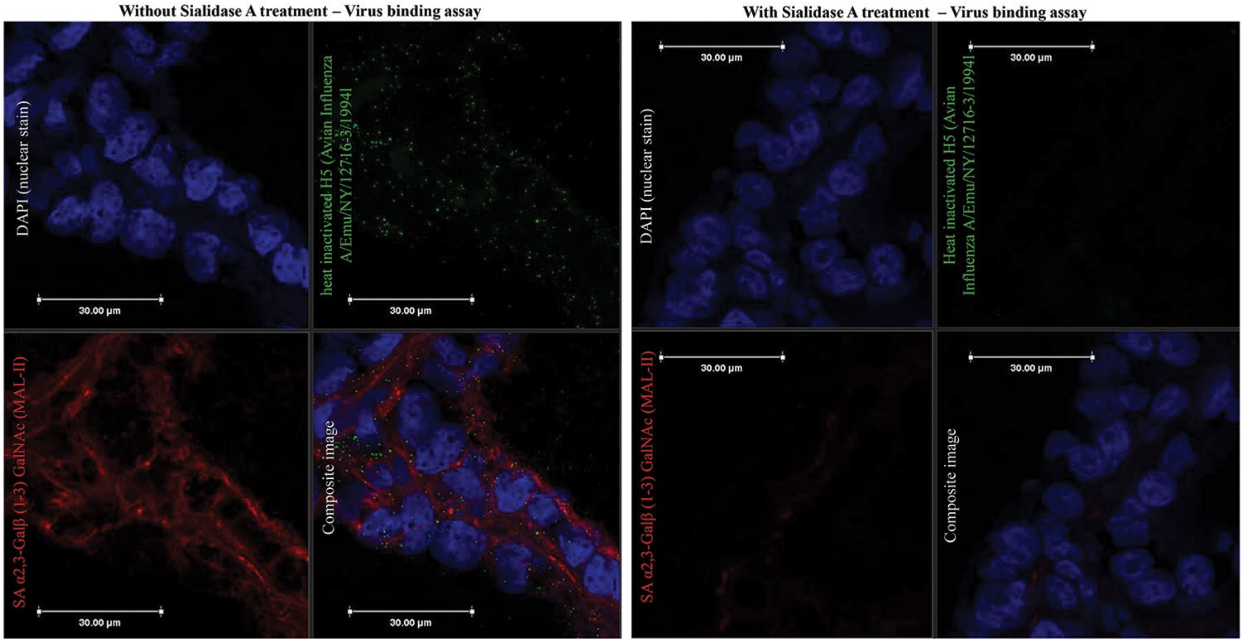

Appendix Figure 3. MAL-II lectin (red) and IAV-H5 (green) virus binding assays on normal healthy bovine mammary gland treated with/without sialidase-A. Scale bar = 30 µm.

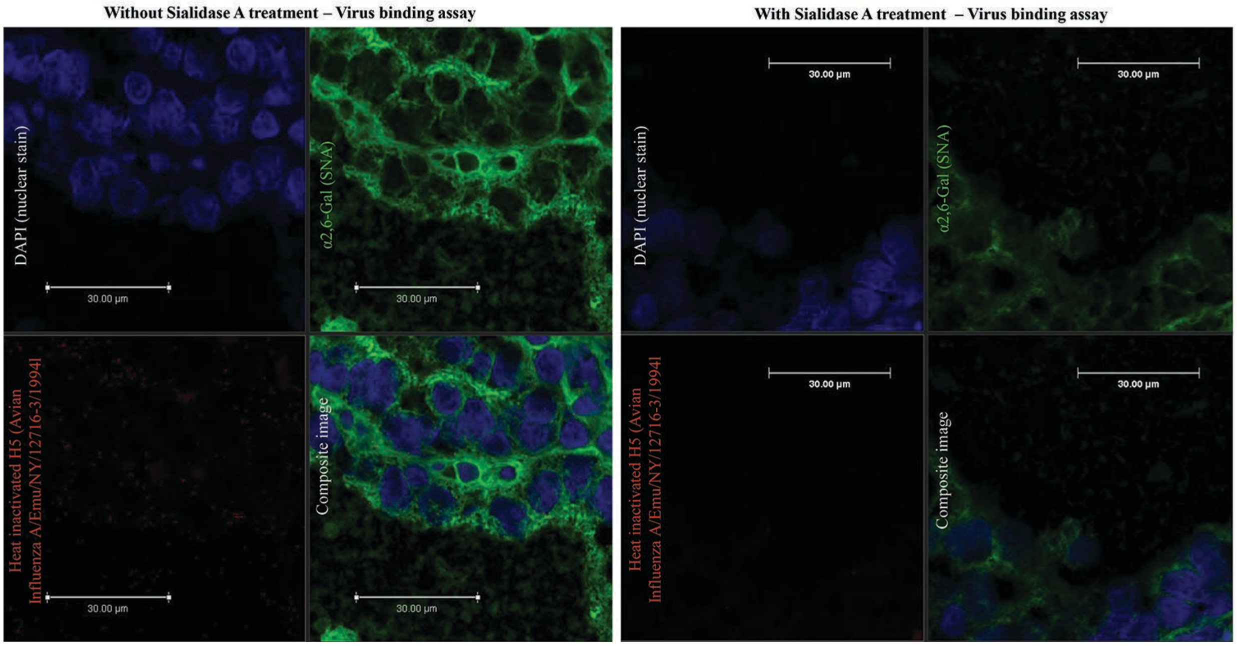

Appendix Figure 4. SNA lectin (green) and IAV-H5 (red) virus binding assays on normal healthy bovine mammary gland treated with/without sialidase-A. Scale bar = 30 μm.
